# Isolation and identification of a bovine viral diarrhea virus from sika deer in china

**DOI:** 10.1186/1743-422X-8-83

**Published:** 2011-02-25

**Authors:** Yugang Gao, Shijie Wang, Rui Du, Quankai Wang, Changjiang Sun, Nan Wang, Pengju Zhang, Lianxue Zhang

**Affiliations:** 1 College of Traditional Material Medicine, Jinlin Agriculture University, Changchun, 130118, PR China; 2 College of Traditional Material Medicine, Jinlin University, Changchun, 130118, PR China; 3 China Institute of Veterinary Drug Control, Beijing, PR China; 4 School of Life Science, Sichuan University, Chengdu, 610064, PR China

## Abstract

**Background:**

Bovine viral diarrhea virus (BVDV) infections continue to cause significantly losses in the deer population. Better isolation and identification of BVDV from sika deer may contribute significantly to the development of prophylactic therapeutic, and diagnostic reagents as well as help in prevention and control of BVDV. However, isolation and identification of BVDV from sika deer is seldom reported in literature. In this study, we collected some samples according to clinical sign of BVDV to isolation and identification of BVDV from sika deer.

**Results:**

we isolated a suspected BVDV strain from livers of an aborted fetus from sika deer in Changchun (China) using MDBK cell lines, named as CCSYD strain, and identified it by cytopathic effect (CPE), indirect immunoperoxidase test (IPX) and electron microscopy(EM). The results indicated that this virus was BVDV by a series of identification. The structural proteins E0 gene was cloned and sequenced. The obtained E0 gene sequence has been submitted to GenBank with the accession number: FJ555203. Alignment with other 9 strains of BVDV, 7 strains of classical swine fever virus (CSFV) and 3 strains of border disease virus(BDV) in the world, showed that the homology were 98.6%-84.8%, 76.0%-74.7%, 76.6%-77.0% for nucleotide sequence, respectively. The phylogenetic analysis indicated that new isolation and identification CCSYD strain belonged to BVDV1b.

**Conclusion:**

To the best of our knowledge, this is the first report that BVDV was isolated and identified in sika deer. This current research contributes development new BVDV vaccine to prevent and control of BVD in sika deer.

## Background

Bovine viral diarrhea virus, a single-stranded RNA is found in cattle and other ruminants worldwide [1-4]. The presence of BVDV in other domestic species such as sheep or wild species such as whitetail deer might be relevant to the epidemiology other disease in cattle [5]. The BVDV infections range from clinically in apparent infections to severe disease involving one or more organ systems. Historically, BVDV was associated with digestive tract disease including high mortality. Currently, BVDV is associated more frequently with respiratory disease and fetal infections [2]. Raise skia deer already had hundreds years at present artificially in china and farmed populations had reached hundreds of thousands in recent year. However, bovine viral diarrhea (BVD) caused significantly losses in the deer population. It was reported that infections rates of BVDV for young deer reached 60%~86.7% in some areas of china in recently years [6], which caused economic losses to sika deer industry due to the high morbidity and fetal infections associated with the disease. Thus, the isolation and identification of BVDV from sika deer, which is fundamental to prevent and control of BVDV in sika deer, becomes an urgent task to many researchers.

The Bovine viral diarrhea virus (BVDV) belongs to the genus *pestivirus *within the family *Flaviviridae*. BVDV is closely related to the classical swine fever virus (CSFV) and the ovine border disease virus (BDV) [7]. The pestiviral genome consists of a single stranded positive sense RNA with a length of about 12.3 kb. It contains one larger open reading frame (ORF), which is flanked by nontranslated regions (NTR) on both genome termini. The single ORF is translated into one polyprotein, which is co-and post- translationally processed into the mature proteins N^pro^, C, E0 (gp48, also named E^rns^), E1, E2, NS2/3, NS4a, NS4b, NS5a and NS5b by viral and cellular proteases [8-10]. E0 protein, the main structural protein of BVDV, plays a very important role in inducing protective immunoreaction against BVDV and diagnosing virus [2].

Although BVDV from the skia deer has been seriously concerned, there are only a few articles with respect to the prevalence of BVDV investigations and BVDV clinical sign [6], Particular, lacking in articles with regard to the molecular virology, gene sequences and genetically engineering vaccine of local BVDV isolates in China due to BVDV from skia deer without isolation and identification. The purpose of our study was isolation and identification BVDV from skia deer by a series of methods, which contributes this disease control.

## Results

### Isolation and identification

**Figure 1:**
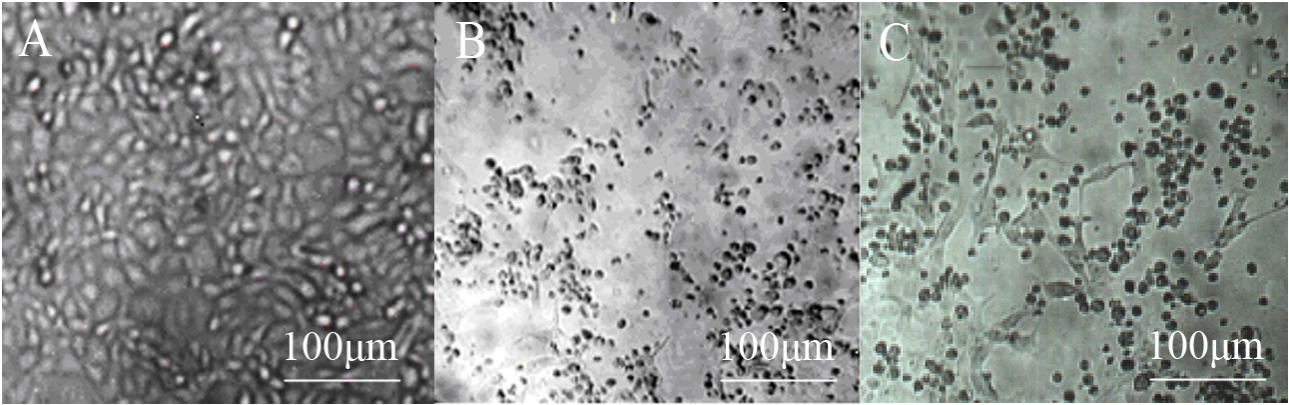
**The CPE of BVDV**. A: The MDBK cell as negative control; B: The CPE of C_24_V strain as positive control. C: The CPE of CCSYD strain.

**Figure 2:**
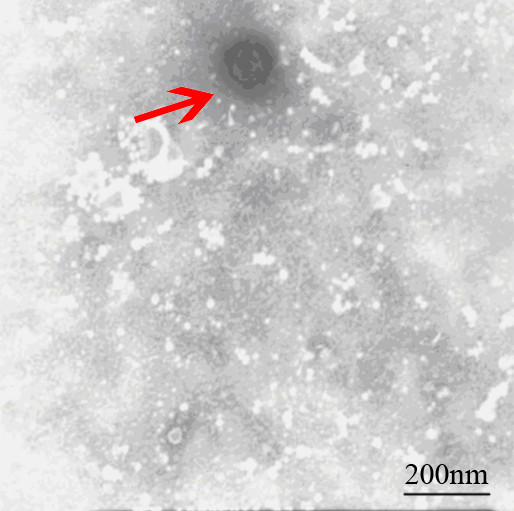
**Negatively stained BVDV**.

The significant cytopathic effects (CPE) were observed in MDBK cell infected with virus 24h-48h. The MDBK cells generate obvious cell lesion and net between cells in comparison to the control cells, which is consistent with CPE of BVDV on MDBK cell (Figure 1). The IPX assay showed that the well of positive serum appeared red-brown cytoplasmic staining, which suggested that new isolation virus might be BVDV. The negatively stained virus particles extracted from liver were approximately 40 nm-60 nm in diameter when examined by electron microscope (Figure 2), and displayed a typical BVDV morphology.

### Amplification, sequencing and analysis of E0 gene

**Figure 3:**
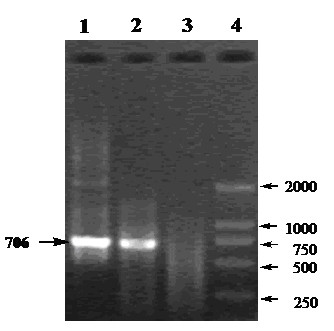
**The amplified products by RT-PCR**. Lan1 and Lan2: E0 gene from infected MDBK cell; Lan3: MDBK cell as negative control.

The MDBK cell infected with CCSYD were positive by the RT-PCR assays, and the expected sizes of the PCR products, 706 bp for E0 of CCSYD, were observed as clear electrophoretic band (Figure 3). The obtained E0 genes segment by sequencing has been deposited in GeneBank under accession No. FJ55520. Alignment with other 9 strains of BVDV, 7 strains of CSFV and 3 strains of BDV in the world, showed that the homology were 98.6%-84.8%, 76.0%-74.7%, 76.6%-77.0% for nucleotide sequence, respectively (Table 1), which shows that there is no significant deviation of CCSYD E0 with conventional BVDV.

**Table 1:** Homology of E0 gene sequence of different RHDV isolates

	C_24_V	R1935	naqdl	SD-1	Bega	Y546	CCSYD	VEDEVAC	ILLC	OSLOSS	ALD	GPE	Brescia	SM	C	LN9912	JL	BD31	BDVX818	C413
C_24_V		99.5	91.5	92.7	88.4	88.6	84.9	84.6	85.6	85.2	75.5	75.4	74.5	75.5	76.0	75.8	75.2	77.1	77.0	76.6
R1935	0.6		91.5	92.7	88.6	88.9	85.2	84.8	85.8	85.2	75.5	75.4	74.6	75.5	76.0	75.8	75.3	77.1	77.0	76.5
naqdl	11.8	11.8		91.5	87.7	87.8	86.2	85.5	87.0	85.6	75.6	75.8	75.2	75.3	75.1	74.8	75.1	77.8	77.5	76.9
SD-1	10.0	10.0	11.8		88.1	88.6	84.8	84.7	85.7	85.7	75.1	74.9	74.0	74.9	75.4	75.2	74.7	78.9	78.0	76.7
Bega	16.9	16.5	18.0	17.4		95.1	85.8	85.8	87.4	86.4	74.8	74.9	74.9	74.9	74.5	74.5	76.3	77.7	77.6	76.9
Y546	16.6	16.2	17.9	16.5	6.5		85.5	85.4	86.7	86.5	74.5	74.6	74.7	74.4	73.9	73.9	76.2	79.1	78.2	76.3
CCSYD	22.8	22.4	20.5	23.2	21.2	21.8		98.6	92.7	95.1	76.0	76.1	75.1	75.8	74.7	74.9	75.5	76.6	76.7	77.0
VEDEVAC	23.5	23.1	21.8	23.4	21.2	22.1	1.8		93.4	95.8	76.2	76.3	75.1	76.0	74.9	74.9	75.8	76.9	76.7	77.4
ILLC	21.7	21.3	19.2	21.5	18.6	19.9	10.0	9.0		92.6	76.0	76.2	75.3	76.0	75.5	75.5	76.7	77.4	77.1	77.8
OSLOSS	22.6	22.6	21.6	21.5	20.2	20.0	6.5	5.5	10.3		76.1	76.2	75.2	76.0	75.2	75.2	76.1	77.3	76.6	77.1
ALD	40.9	40.9	40.8	42.3	42.7	43.6	40.1	39.4	39.9	39.6		99.3	94.7	98.6	96.6	96.6	88.2	79.2	79.1	77.6
GPE	41.2	41.2	40.4	42.6	42.4	43.2	39.8	39.1	39.3	39.3	0.9		94.1	97.9	96.1	96.1	88.5	79.4	79.4	77.7
Brescia	43.2	42.9	41.6	44.6	42.1	42.6	42.0	41.9	41.3	41.5	7.1	8.0		94.4	92.8	93.3	87.4	78.3	78.0	76.8
SM	40.9	40.9	41.6	42.6	42.3	43.7	40.5	39.8	39.8	39.7	1.8	2.7	7.5		96.4	96.6	88.3	79.2	79.1	77.3
C	39.8	39.8	42.2	41.4	43.7	45.1	43.3	42.5	41.0	41.8	4.4	5.2	9.8	4.7		99.5	86.8	78.9	78.7	77.8
LN9912	40.3	40.3	42.7	41.9	43.6	45.1	42.6	42.5	40.9	41.7	4.4	5.2	9.2	4.4	0.6		86.8	78.9	78.7	77.6
JL	41.6	41.3	42.0	43.0	38.8	39.1	41.0	40.3	38.1	39.4	17.3	16.7	18.5	17.0	19.7	19.7		78.5	79.0	76.7
BD31	37.2	37.2	35.8	33.5	36.0	33.0	38.3	37.6	36.6	36.8	33.7	33.5	35.6	33.8	34.5	34.5	34.8		92.3	77.4
BDVX818	37.4	37.4	36.5	35.4	36.3	35.0	38.1	38.2	37.2	38.5	33.9	33.4	36.4	33.9	34.9	35.0	33.9	10.6		76.7
C413	38.4	38.7	37.8	38.2	37.8	39.1	37.2	36.5	35.5	36.9	36.2	35.9	37.9	37.0	35.7	36.2	37.9	36.7	38.3	

### Phylogenetic analysis

**Figure 4:**
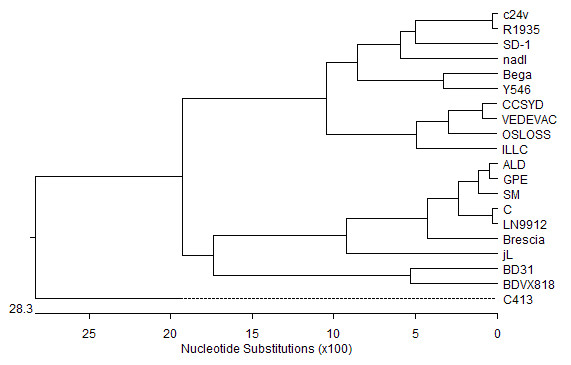
**Phylogenetic analysis of E0 protein sequence of different BVDV isolates**.

To better understand the relationship of CCSYD to other 9 strain of BVDV, 7 strains of CSFV and 3 strains of BDV variants co-circulating in the word, genetic sequences of *Pestivirus *from cattle, swine and ovine in GenBank were used to construct phylogenetic trees. Figure 4 clearly showed that E0 genes of the CCSYD belonged to BVDV1b.

## Discussion

In the present study, CCSYD strain was isolated from skia deer in Changchun city. By employing a series of biochemical and biophysical methods, we have firstly identified that CCSYD might be BVDV. The BVDV can be classified into biotypes and genotypes [2]. Biotypes are based on the presence or absence of visible CPE in infected cell cultures, cytopathic (CP) or noncytopathic (NCP) [2]. Genotype classification is based on divergence in the viral genome sequences revealed by phylogenetic analysis [11-15]. Based on phylogenetic comparison, the virus can be classified into two genotypes: BVDV1 and BVDV2. Whereas BVDV1 has a world-wide distribution, BVDV2 appears to be highly prevalent only in North America [13,16] and relatively rare in other continents [15,17]. Moreover, recently the BVDV1 and BVDV2 genotypes have been further divided into subgenotypes BVDV1a, BVDV1b, BVDV2a, and BVDV2b in North American [18,19]. Genetic and phylogenetic analysis showed that the virus belonged to BVDV1b. Similar strains contain the VEDEVAC strain isolated Hungary, with 98.7% homology for amino acid. Moreover, we have also successfully cloned NS2/3 genes of CCSYD strain, with 100% homology for amino acid and NS2/3 genes of VEDEVAC strain, which further showed that CCSYD strain and VEDEVAC strain belonged to BVDV1b.

The protection conferred by conventional inactivated BVDV vaccines is strongly correlated with genetically and antigenically of specific BVDV strain. In addition to, an increasing frequency of skia deer with elevated antibody titers to BVDV suggests that exposure to field strains has not been diminished despite the use of both management and vaccine [6]. This suggests that current vaccine may be inadequate in conferring skia deer protection against the acute disease in skia deer. Design of efficacious vaccines must be based on specific BVDV strain about the immune responses critical to development of protection. Hence, it is appropriate to guide the selection of the vaccine strain according to the specific BVDV strain from local area and similar strain, which depend on further studies of BVDV from skia deer distribution and identification. There is currently no commercial skia deer BVDV vaccine used in China, and therefore, it is important to select the vaccine candidate strain from control BVD from skia deer. Inactivated CCSYD strain by a certain method might be employed in preventing and counterchecking the skia deer BVD in Jilin province. Moreover, selected E_0 _protein of CCSYD construction a DNA vaccine might induce cellular immune response and antibody responses specific for BVD from skia deer. Therefore, isolation and identifcation of a skia deer bovine viral diarrhea virus (CCSYD) contributes development new BVDV vaccine to prevent and control of BVDV in sika deer.

## Conclusions

The present study described the isolation and identifcation of a skia deer bovine viral diarrhea virus (CCSYD) isolated from sick skia deer for the first time in China. This study provided a detailed analysis of the genetic and evolution of CCSYD which is likely to be helpful to guide efficient diagnostic, preventive and control strategies against BVD from skia deer in China.

## Materials and methods

### Virus isolation

Field samples came from skia deer at field of Changchun (China), which might infected with BVDV according to clinical sign such as diarrhea and miscarriage and stillbirth. Skia deer liver was collected from an aborted fetus deer. A total of 1 g of fresh liver tissue was homogenized in 4 ml of phosphate-buffered saline (PBS, PH 7.2) and then repeated freeze thawed 3 times, and centrifuged at 5000 rpm for 30 min. A certain liver extract was then inoculated onto MDBK monolayer cultures, 25 cm flasks with a 1 ml inoculum (1:10 dilution of original samples) and the total volume was 5 ml. The MDBK cultures were observed for 6 days with presence or absences of CPE recorded. The cultures were frozen at 70°C, thawed and supernatants collected after centrifugation with subsequent storage at 70°C. Uninoculated cultures were included as negative controls and inoculated C_24_V as positive control.

### Identification of virus

BVDV was detected by indirect immunoperoxidase test (IPX) and electron microscopy technique (EM). For IPX, the test was carried out on 96-well plates using low-passage MDBK cells. Serum (20 μl) was added to each of four wells before the addition of 100 μl of cell suspension. Positive and negative control sera were run on each plate. The test plates were incubated for 4 days in 5% CO_2_, 37°C. Plates were fixed and dried, then stained with immunoperoxidase as described by Meyling [20], using a polyclonal bovine anti-BVDV serum (BVD virus positive control serum, China) to detect the virus. The presence of red-brown cytoplasmic staining in any of the wells exposed to the specific anti-BVDV antibody denoted a positive result. For electron microscopy technique (EM), chloroform (1/10 of the volume of the liver extract) was added into the liver extract, and then the liver extract) was added into the liver extract, and then the mixture was incubated for 30 min at 4°C and then centrifuged at 12000 rpm for 30 min. The precipitate was resuspended in 0.005 M moderate phosphate buffered saline (PBS; PH 7.2), and negatively stained with 2% phosphotungstic acid. The specimens were examined with a transmission electron microscope (Hitachi-8100, Japan) at 80 kV.

### PCR amplification and sequencing

Total RNA was isolated from infected MDBK cell using TRIzol reagent (Invitrogen China) according to the manufacturer's protocol and as described in the online supplement. In short, 200 μl infected MDBK cell was incubated with 1 ml TRIzol for 5 min at room temperature (RT). Cell debris was removed by centrifugation (12,000 × g at 4°C for 10 min) and 0.4 ml chloroform was added. After vortexing the mix was incubated for 5 min at RT. The phases were separated by centrifugation (12,000 × g at 4°C for 15 min) and the aqueous phase was transferred to a new tube. 0.6 × volume of isopropyl alcohol and a 0.1 × volume of 3 M sodium acetate were added to this aqueous phase and incubated for 10 min at 4°C. The precipitated RNA was pelleted by centrifugation (12,000 × g at 4°C for 15 min) and after the removal of the supernatant the RNA pellet was washed twice with 70% ethanol. After drying, the RNA was resuspended in 30 μl DEPC-treated water. Using mRNA as template, single-stranded cDNAs were generated by Superscript II reverse transcriptase (Invitrogen) according to the manufacturer's directions. The E0 primer sequences were as follows: sense prime: 5'-CCGGATCCACCATGGAAAACATAACACAGTGG-3'; anti-sense prime: 5'- GCCTCGAGTTAAGCGTATGCTCCAAACCACGT -3'. The PCR conditions were 94°C for 3 min, followed by 30 cycles of DNA amplification (45s at 94°C, 1 min at 61°C, and 1 min 30s at 72°C) and 8 min incubation at 72°C. PCR products were separated by electrophoresis at a constant voltage (2 V/cm) in a 1.2% (w/v) agarose gel. The full-length E0 gene subjected to DNA sequencing.

### Analysis of sequence

The sequence data were analyzed with computer programs such as DNAMAN and DNASTAR. Phylogenetic analysis was done by the distance-based Neighbor-joining method using software EGA 4.1. (DNAStar Inc.).

## Abbreviations

BDV: Border Disease Virus; BVD: Bovine viral diarrhea; BVDV: Bovine Viral Diarrhea Virus;CPE: Cytopathic Effects; CSFV: Classical Swine Fever Virus; DNA: Deoxyribonucleic Acid; EM: Electron Microscopy; IPX: Indirect Immunoperoxidase; MDBK: Bovine Kidney; PCR: Polymerase Chain Reaction; RNA: Ribonucleic Acid.

## Competing interests

The authors declare that they have no competing interests.

## Authors' contributions

YG and PZ participated in the design and conducted the majority of the experiments in the study and drafted the manuscript. RD and SW contributed to the interpretation of the findings and revised the manuscript. QW and NW edited the manuscript. CS performed analyses of data. All authors read and approved the final manuscript.
